# Correlation between real sieving coefficient determined using novel lab-scale module and pore structure of hemodiafiltration membranes

**DOI:** 10.1007/s10047-026-01577-4

**Published:** 2026-07-30

**Authors:** Mizuki Ichikawa, Makoto Fukuda, Takuma Morikawa, Kousei Takeuchi, Kiyotaka Sakai

**Affiliations:** 1 Graduate School of Biology-Oriented Science and Technology, Kindai University, 930 Nishimitani, Kinokawa, Wakayama, 649-6493 Japan; 2 Department of Biomedical Engineering, Kindai University, 930 Nishimitani, Kinokawa, Wakayama, 649-6493 Japan; 3 Clinical Engineering, Suita tokushukai Hospital, 21-1 Senrioka-Nishi, Suita, Osaka, 565-0814 Japan; 4 Professor Emeritus of Chemical Engineering, Waseda University, 3-4-1 Okubo, Shinjuku-ku, Tokyo, 169-8555 Japan

**Keywords:** Hollow fiber membrane, Sieving coefficient dextran, Hemodiafiltration, FE-SEM

## Abstract

The novel lab-scale hollow fiber membrane module was fabricated to determine the apparent sieving coefficient (${SC}_{obs}$) of commercial hemodiafiltration membranes, MFX-SW eco (polyethersulfone, Sample A) and NVF-P (polysulfone, Sample B), using the aqueous solution of dextran with a broader molecular weight distribution. All the molecular weight cut-off curves plotted as ${SC}_{obs}$ versus filtration flow rate (*Q*_*F*_) exhibited smooth profiles without intersecting each other, showing distinct variations depending on *Q*_*F*_ across a wide range of molecular weights. Field emission scanning electron microscopy (FE-SEM) observations revealed that the equivalent pore diameters of samples A and B were nearly identical at 23.0 ± 15.5 nm and 23.3 ± 15.5 nm, respectively. Although the surface porosity of sample A (14.5 ± 3.9%) was higher than that of sample B (10.2 ± 3.2%), the difference was not statistically significant. These numerical data were in excellent agreement with the qualitative morphological findings obtained from the FE-SEM images. Furthermore, it is suggested that the subtle structural differences between the two membranes are also reflected in the relationship between the real sieving coefficients (${SC}_{real}$) calculated by using ${SC}_{obs}$ and the equivalent pore diameters. Since the lab-scale modules are fabricated relatively easily by hand, this methodology is extremely valuable to rigorously define membrane performance.

## Introduction

Since Dow Chemical Co. initiated the mass production of hollow-fiber blood purifiers in 1968, blood purification technology has advanced remarkably. Progress in blood purification membranes, driven by industrial chemistry innovations such as polymer synthesis and hollow fiber fabrication techniques, has transformed the field into a major medical device industry. Furthermore, the establishment of microscopic observation techniques and membrane structural analysis methods, alongside the evolution of membrane transport theories based on theoretical and quantitative approaches, has propelled this field forward. Crucial innovations in blood purification membranes were marked by the introduction of hollow fiber configurations and polysulfone membranes [1].

In recent years, in the field of clinical artificial kidneys, driven by rapid global market expansion and globalization, competition in the development and commercialization of artificial kidneys is intensifying utilizing polysulfone (PS) or polyethersulfone (PES) membranes blended with polyvinylpyrrolidone (PVP) as the biocompatible material [2–4]. Under certain circumstances, advanced research and development for social implementation are strongly required, as well as further improvement and stabilization of product design and manufacturing quality. Moreover, the number of patients undergoing hemodiafiltration (HDF) therapy in Japan has increased rapidly, accounting for 63.3% of all treatment modalities as of 2024 [5]. Compared with conventional hemodialysis (HD) therapy, the total performance of HDF therapy is heavily influenced by operating conditions, such as the filtration flow rate, transmembrane pressure (TMP), and their variations over time. Therefore, it is essential to appropriately define fundamental performance characteristics, such as the molecular weight cut-off properties of the hemodiafiltration membrane, during the design stage and to strictly control manufacturing conditions and quality during production. Consequently, a more detailed understanding of the relationship between the performance characteristics (real sieving coefficient) of hemodiafiltration membranes and their pore structures is required [1].

Mineshima et al. [6] measured the apparent sieving coefficients of industrial-scale modules using commercial dialyzers and aqueous dextran solutions. Although they demonstrated characteristic dextran sieving coefficient curves for each membrane type, it requires the use of full-scale commercial modules, making it difficult to evaluate tiny amounts of membrane samples. Furthermore, because the apparent sieving coefficient is affected by blood flow and pass within the module, the real sieving coefficient must be determined to correlate the membrane structure with the sieving properties [7, 8]. Meanwhile, in the field of gas separation membranes, lab-scale hollow fiber membrane modules have been fabricated to verify the performance equivalence between lab-scale and industrial-scale membrane systems [9].

Therefore, the present study aims to advance these pioneering studies by fabricating the original lab-scale module and employing an aqueous solution of dextran with a broader molecular weight distribution. This approach attempts to efficiently and cost-effectively measure the precise apparent sieving coefficients of commercial hemodiafiltration membranes. Based on these measurements, the real sieving coefficients are calculated to elucidate their relationship with the membrane pore structures.

## Experimental

### Materials

Two commercial hemodiafiltration (HDF) membranes widely utilized with similar characteristics were evaluated: MFX-15SW eco® (Sample A; polyethersulfone membrane; Nipro Corp., Osaka, Japan) and NVF-21P (Sample B; polysulfone membrane; Toray Industries, Inc., Tokyo, Japan). Table 1 summarizes the technical specification data for each sample.

**Table 1:** Technical data of hollow fiber hemodiafiltration membranes tested

Membrane	Manufacturer	Membrane materials	Inner diameter [μm]	Wall thickness [μm]	Sterilization method	Product condition
Sample A MFX®-15SWeco Lot No. 23L28F	Nipro Co., Ltd	Polyethersulfone, hydrophilized by blending poly(vinylpyrrolidone) (PVP)	215	45	γ-ray	Dry
Sample B TORAYLIGHT®HDF NVF-21P Lot No. 50362010	Toray Co., Ltd	Polysulfone, hydrophilized by blending poly(vinylpyrrolidone) (PVP) and NV polymer	200	40	γ-ray	Moist

Although the base membrane materials differ, both samples incorporate PVP as a hydrophilic agent to suppress blood-component damage and fouling on the membrane surfaces. Furthermore, the novel hydrophilic polymer (NV polymer) is immobilized on the inner surface of sample B, whose adsorbed water enhances biocompatibility [10, 11]. In addition, they differ in their product conditions.

### Fabrication of novel lab-scale hollow fiber membrane module

**Fig. 1:**
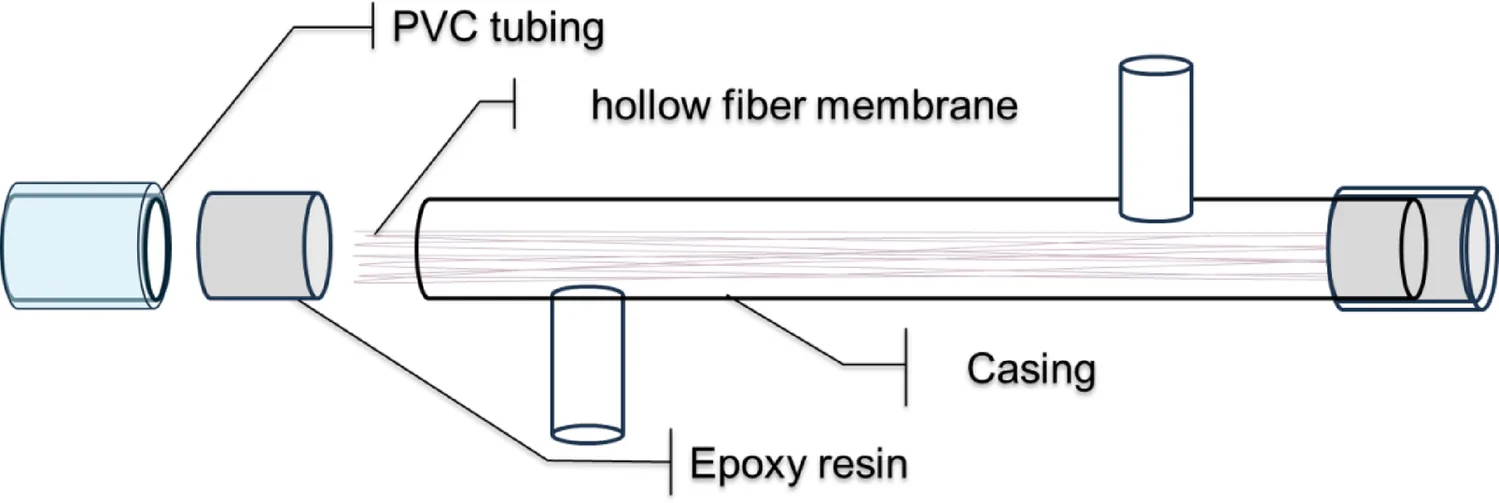
Configuration of the lab-scale hollow fiber membrane module. The unit is comprised of a bundle of hollow fiber membranes within a housing of 5 mm inner diameter. The overall and effective lengths of a module are 15 cm and 11 cm, respectively

The configuration of the lab-scale hollow fiber membrane module is illustrated in Fig. 1. Hollow fiber membranes (effective length: 11 cm, number of fibers: 135 (sample A) or 150 (sample B), inner surface area: approximately 0.01 m^2^) were loaded into a plastic housing with a length of 15 cm and an inner diameter of 5 mm. The housing, hollow fiber membranes, and poly (vinyl chloride) tubes were fixed using an adhesive (a two-component epoxy resin, Quick Mender; Konishi Co., Ltd., Osaka, Japan). After the adhesive had been cured, both ends of the module were cleaved to open the cross-sections of the hollow fibers. These sections were then inspected to verify that the fibers were uniformly distributed, that there were no unfilled areas or gaps in the adhesive, and that the fibers were not collapsed or obstructed. A leak test was subsequently performed by pressurizing the inner lumen side of the hollow fiber membranes. The production yield of defect-free, intact lab-scale modules suitable for evaluation was over 80%, and apparent sieving coefficient (${SC}_{obs}$) was measured using lab-scale modules.

Fiber density (*f*_*D*_) (%) is the proportion of the total volume of hollow fibers in the internal volume of a housing and is calculated by Eq. (1):

1 $${f}_{D}=\frac{N{ (d+2\Delta x)}^{2}}{{{D}_{h}}^{2}} \times 100 (\%)$$

where *N* is the number of fibers (-), *d* is the inner diameter of a hollow fiber membrane (m), *Δx* is the wall thickness of a hollow fiber membrane (m) and *D*_*h*_ is the inner diameter of a housing (m).

Inner surface area (m^2^) is calculated by Eq. (2):

2 $$A=d\pi NL ({m}^{2})$$

where *L* is the effective length of a hollow fiber (m).

### Measurement of apparent sieving coefficient utilizing the lab-scale hollow fiber membrane module and calculation of the real sieving coefficient

**Fig. 2:**
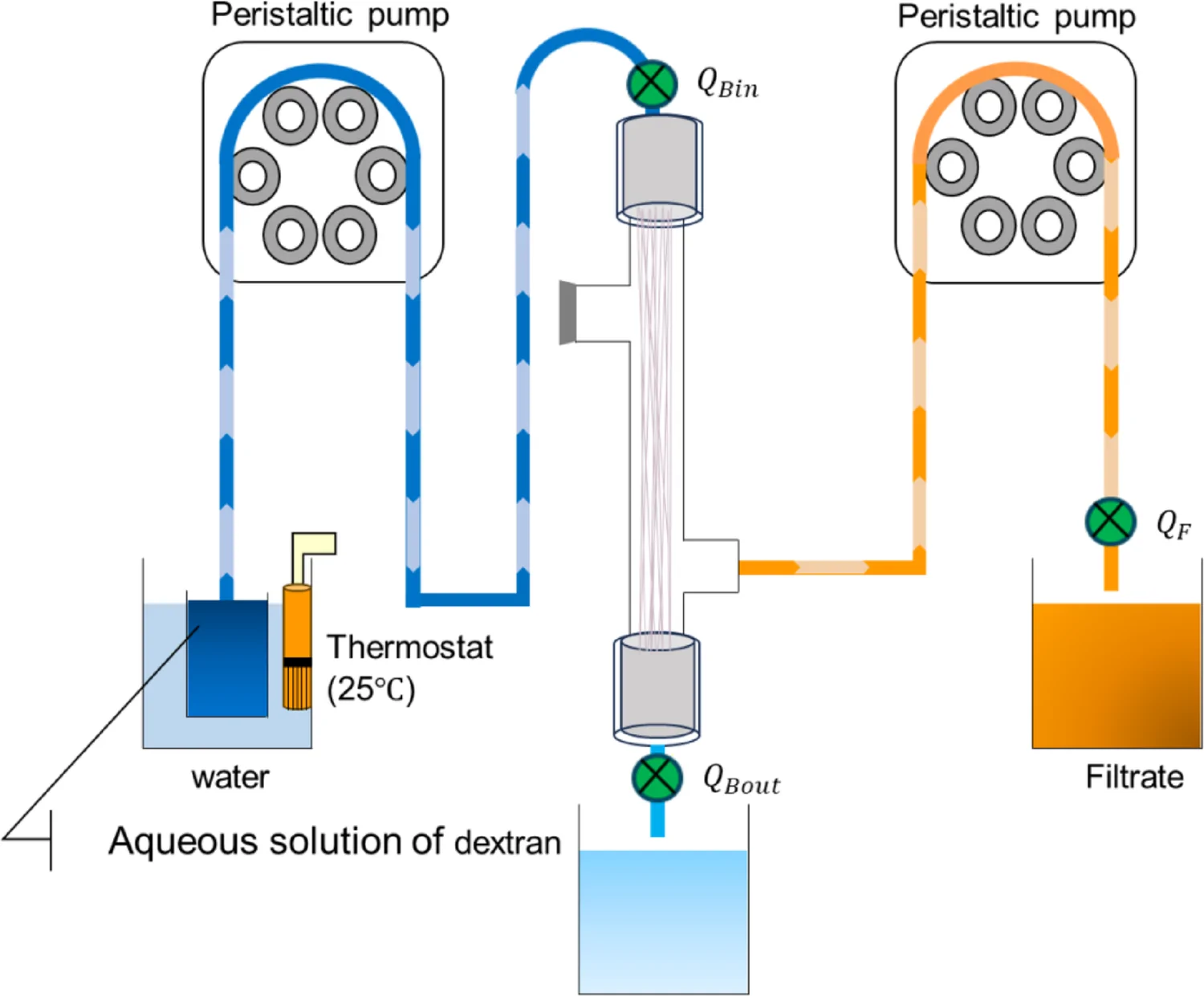
**a** Schematic diagram of the SC-measurement circuit. The apparatlus is comprised of a lab-scale module connected to a 2 mm I.D. circuit. Peristaltic pumps maintain the flow

The experimental apparatus for sieving coefficient measurement is shown in Fig. 2. The circuit, consisting of tubing with an inner diameter of 2 mm, was connected to a peristaltic pump (AC-2120; ATTO Corp., Tokyo, Japan) and the lab-scale module. A dextran aqueous solution was perfused through the circuit at a constant temperature of 25 °C for at least 10 min. The steady-state waiting time was set in accordance with the previous study [12]. After verifying that a steady state had been established, fluid sampling was performed at the blood inlet, blood outlet and filtrated outlet. The operational parameters for the lab-scale module were scaled appropriately, with the $\mathrm{b}\mathrm{l}\mathrm{o}\mathrm{o}\mathrm{d}-\mathrm{s}\mathrm{i}\mathrm{d}\mathrm{e} \mathrm{i}\mathrm{n}\mathrm{l}\mathrm{e}\mathrm{t} \mathrm{f}\mathrm{l}\mathrm{o}\mathrm{w} \mathrm{r}\mathrm{a}\mathrm{t}\mathrm{e} ({Q}_{BI})$ of 1.38 mL/min and filtration flow rate (${Q}_{F}$) ranging from 0.10 to 0.62 mL/min, to be matched for the operating conditions of a full-scale module—specifically a ${Q}_{BI}$ of 200 mL/min and a ${Q}_{F}$ of 10–60 mL/(min·m^2^).

The dextran solution, with a total concentration of 3.0 g/L, was prepared using six types of dextran with different weight-average molecular weights (MW: 1200; 6000; 15,000–20,000; 40,000; 56,000; and 222,000; Sigma-Aldrich Co. LLC, St. Louis, MO, USA). The refractive index of the sampled solutions was measured via gel permeation chromatography (GPC) using a high-performance liquid chromatography system (HLC-8420GPC; Tosoh Corporation, Tokyo, Japan). A molecular weight calibration curve was constructed for each prepared solution. The appropriate column for the dextran aqueous solutions used in this study was selected, and after establishing the analytical conditions, calibration curves were plotted for each aqueous solution, making it possible to accurately measure the concentration of each molecular weight with a wide range of molecular weights. The molecular weight of the solute in the sample was derived from the retention time, and the concentration (*C*) was determined from the refractive index. ${SC}_{obs}$ was calculated using Eq. (3):

3 $$ SC_{obs} = \frac{{C_{F} }}{{\frac{{C_{Bin} + C_{Bout} }}{2}}} \left( - \right) $$

Data obtained from the samples were statistically analyzed using a two-way analysis of variance (two-way ANOVA, *p* < 0.05). Furthermore, the real sieving coefficient (${SC}_{real}$) was derived from the measured ${SC}_{obs}$ using a methodology based on Eq. (4–8) [7, 8] by defining the experimentally measured values as the ${SC}_{obs}$, and the sieving coefficient independent of ultrafiltration flux and boundary layer mass transfer as the ${SC}_{real}.$ The relationship between ${SC}_{real}$ and the membrane pore structure was validated.

4 $${R}_{real}=\frac{1}{\left(\frac{1-{R}_{obs}}{{R}_{obs}} \frac{1}{{e}^{\frac{{J}_{V}}{k}}}\right)+1} {SC}_{real}=1-{R}_{real}$$

where ${J}_{v}$ is the ultrafiltration flux (*cm/s)*, *k* is the mass transfer coefficient of boundary layer (*cm/s*) which is calculated using Eq. (5) by defining the wall shear rate at the hollow fiber membrane surface as ${\gamma}_{w}$ (*1/s)* and the diffusion coefficient of dextran in water as ${\mathrm{D}}\;{\mathrm{(cm}}^{{2}} {\mathrm{/s)}}$ at 25 °C.

5 $$k=0.816{\left({D}^{2}\frac{{\gamma}_{w}}{L}\right)}^{0.33}\left(\mathrm{c}\mathrm{m}/\mathrm{s}\right)$$

6 $$ \gamma_{w} = \frac{r \cdot \Delta P}{{2\mu L}}\;\left( {1/{\mathrm{s}}} \right) $$

7 $$ D = 8.34 \times 10^{ - 8} \left( {\frac{T}{{\mu MW^{1/3} }}} \right)\left( {{\mathrm{cm}}^{{2}} {\mathrm{/s}}} \right) $$

Furthermore, ${J}_{v}$ is determined using Eq. (8).

8 $$ J_{v} = \frac{{Q_{F} }}{60 \times A}\;\left( {\mathrm{cm/s}} \right) $$

### Observation of the inner surfaces of the hollow fiber membrane utilizing FE-SEM and determination of the pore diameter and surface porosity

Observation of the inner surface morphology and analysis of the pore structure of the hollow fiber membrane were performed in accordance with the procedures described in reference [13]. An FE-SEM (Regulus 8230; Hitachi High-Tech Corporation, Tokyo, Japan) was utilized to observe the inner and outer surfaces of the hollow fiber membrane at an accelerating voltage of 1.0 kV, with observation fields captured at a magnification of 30,000×. An ion sputter coater (MC1000; Hitachi, Ltd., Tokyo, Japan) was employed for platinum (Pt) sputter-coating at 10 mA for 60 s.

Pore areas were measured within the 30,000× magnification observation fields through digital image analysis utilizing ImageJ software (National Institutes of Health, Ver. 1.53, USA). The images were binarized to extract the pore regions. After converting the micrographs into 8-bit grayscale images, the threshold values were adjusted using the "Threshold" function to isolate the pore areas. Additionally, a smoothing filter was applied to reduce image noise and clarify the pore boundaries. The equivalent pore diameters and surface porosities of the images were subsequently calculated based on the measured pore areas. Data obtained from the samples were statistically analyzed using a paired t-test (*P* < 0.05).

## Results and discussion

### Effect of filtration flow rate on the apparent sieving coefficient

**Fig. 3:**
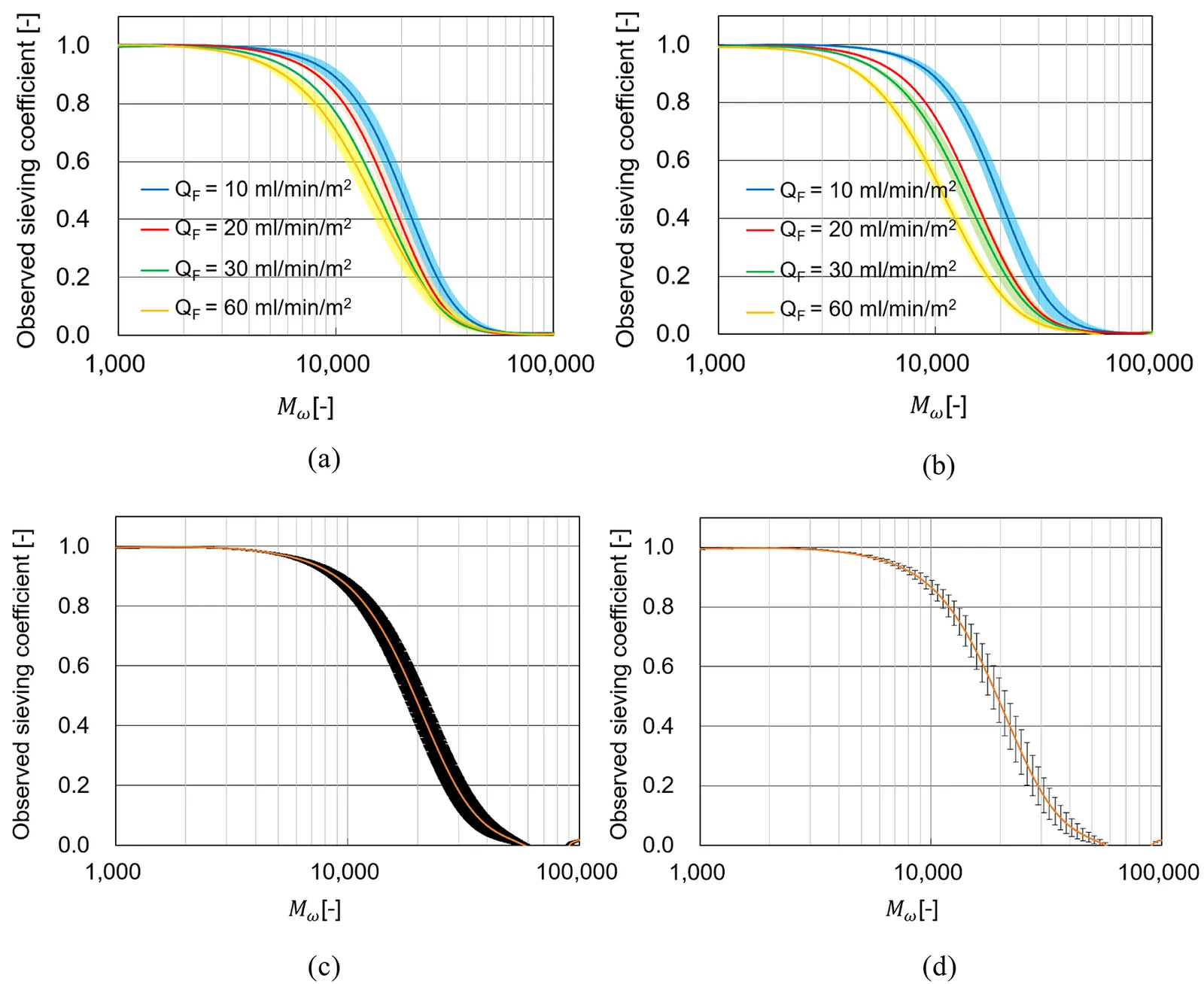
Relationship between weight-average molecular weight (${M}_{\omega }$) and observed sieving coefficient (${S}_{obs}$) at various filtration flow rates (${Q}_{F}$); **a** sample A, **b** sample B, **c** A graph showing the standard deviation of all refractive index data for sample A at Q_F_ = 10 ml/min/m^2^(n = 3) **d** A graph showing the standard deviation when the IR data for sample A at Q_F_ = 10 ml/min/m^2^ is reduced to one-twentieth of its original values (n = 3).

The relationship between the weight-average molecular weight and the measured ${SC}_{obs}$ is illustrated in Fig. 3. The data precision was influenced by several factors, including the precision of the lab-scale module fabrication, the stability of the circulation experiments, the analytical accuracy of the GPC, and the consistency of the dextran solutes. More than six trials were initially conducted from the outset of the study; ultimately, data from three consecutive trials where the values stabilized were selected for analysis. In the figure, the solid lines represent the mean values (n = 3), and the shaded areas represent the standard deviations.

For both sample A and sample B, within the ${Q}_{F}$ range of 10–60 mL/(min·m^2^) (at a blood-side inlet flow rate ${Q}_{BI}$ = 200 mL/min), ${SC}_{obs}$ decreased across all molecular weight ranges as ${Q}_{F}$ increased. This behavior is attributed to the enhanced concentration polarization layer on the inner membrane surface; specifically, as ${Q}_{F}$ increased, the solute concentration on the inner membrane surface $({C}_{Bin},{ C}_{Bout})$ became higher, which subsequently led to a decrease in ${SC}_{obs}$. The molecular weight cut-off curves for all conditions traced smooth trajectories without crossing each other, confirming that distinct sieving coefficient curves were successfully determined across a wide molecular weight range due to the variations in ${Q}_{F}$. Although a larger standard deviation was observed at a ${Q}_{F}$ of 10 mL/(min·m^2^), further improvements are required regarding the sampling protocol after verifying the establishment of the steady state.

All lab-scale modules were utilized on a single-use basis, and no repeated measurements were performed using the same module. Therefore, as exemplified in Fig. 3, the dataset was compiled from a total of at least 24 independent lab-scale modules (n = 3 replicates × 4 filtration flow rate conditions × 2 samples). Consequently, this sample size (n) inherently accounts for module-to-module variability arising from the manual fabrication process.

Figure 3c shows the error bars representing the standard deviations for the original dataset under the conditions of 10 mL/(min·m^2^). Additionally, Fig. 3d shows the error bars in which the data points were subsampled to 1/20th of their original density to enhance visual clarity. These plots represent the standard deviations of the refractive index data obtained from each independent module.

Due to the extremely high density of the refractive index data points, displaying multiple conditions on a single graph could make the standard deviations difficult to discern. To address this, we employed the specific visualization formats shown in Figs. 3 and 6. Consequently, all curves were derived directly from experimental measurements (chromatographic data), and no interpolation procedures, mathematical fitting, or smoothing algorithms were applied.

By expanding the molecular weight range of the dextran solution from the 15,000–60,000 [-] utilized in previous study [6] to a broader range of 1200–222,000 [-], the SC curve in the region corresponding to the molecular weight of albumin (approximately 66.5 kDa) was more clearly defined.

### Relationship between the apparent sieving coefficient and filtration flow rate for specific solute-equivalent molecular weights

**Fig. 4:**
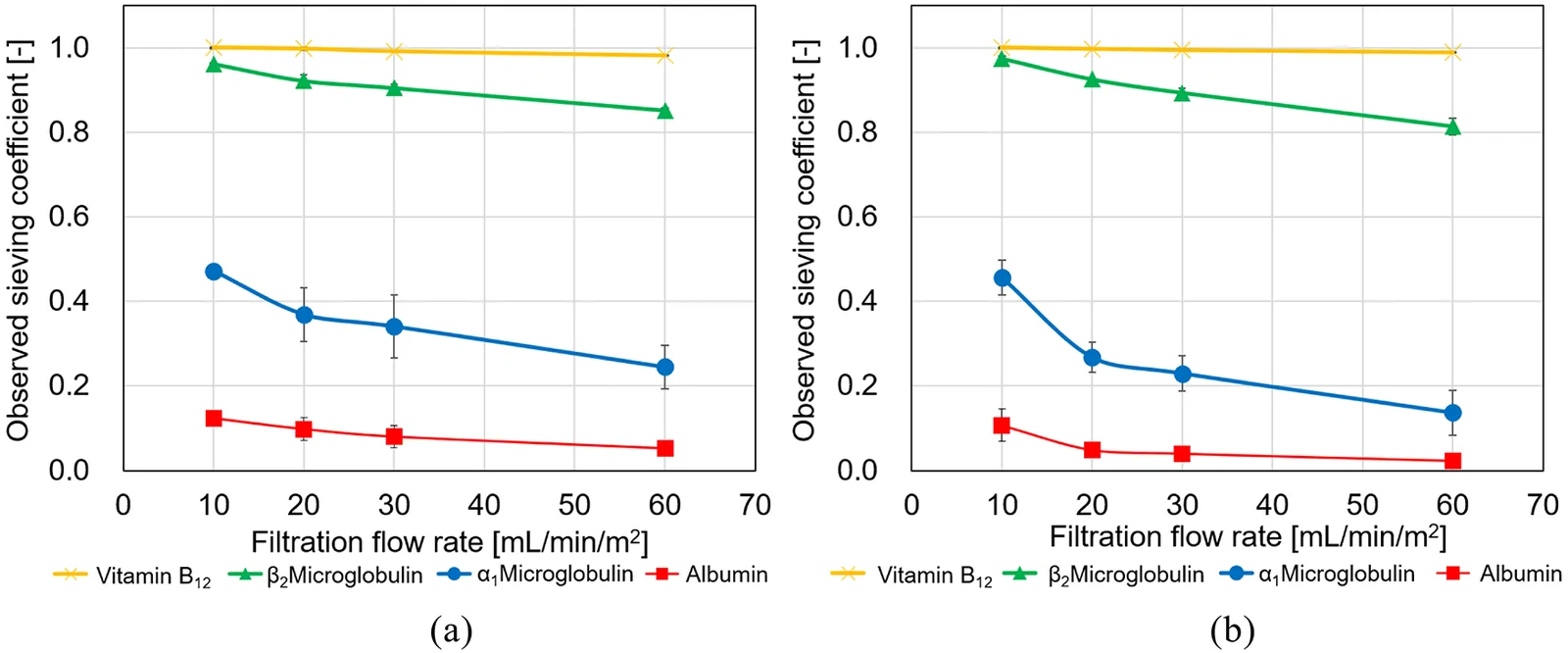
Relationship between filtration flow rate (Q_F_) and observed sieving coefficients (SC_obs_) at molecular weights corresponding to vitamin B_12_, β_2_microglobulin, α_1_microglobulin, and albumin

The relationship between the ${Q}_{F}$ and the ${SC}_{obs}$ for molecular weights corresponding to vitamin B_12_, β_2_microglobulin, α_1_microglobulin, and albumin is shown in Fig. 4. The data presented in Fig. 4 were organized based on the results from Fig. 3.

For the low molecular weight solute corresponding to vitamin B_12_, ${SC}_{obs}$ remained nearly constant even as ${Q}_{F}$ was increased. Conversely, for the larger molecular weights corresponding to α_1_ microglobulin and albumin, a prominent decrease in ${SC}_{obs}$ was observed with increasing ${Q}_{F}$. These results clearly highlighted the molecular-weight-dependent variations in the relationship between ${SC}_{obs}$ and ${Q}_{F}$. Notably, the local minimum in the ${SC}_{obs}$–${Q}_{F}$ curve that had been reported in the previous study [6] was not observed under the experimental conditions of this study in Fig. 4.

Although the ${SC}_{obs}$ may be slightly underestimated at 25 °C compared to 37 °C primarily due to the temperature dependence of fluid viscosity and solute diffusivity, the ${SC}_{real}$ remains essentially unchanged. This is because is defined and calculated as a membrane-specific parameter that doesn’t depend on ultrafiltration flux or mass transfer conditions in the boundary layer.

### FE-SEM image of the inner membrane surface pores and the equivalent pore diameter distribution

**Fig. 5:**
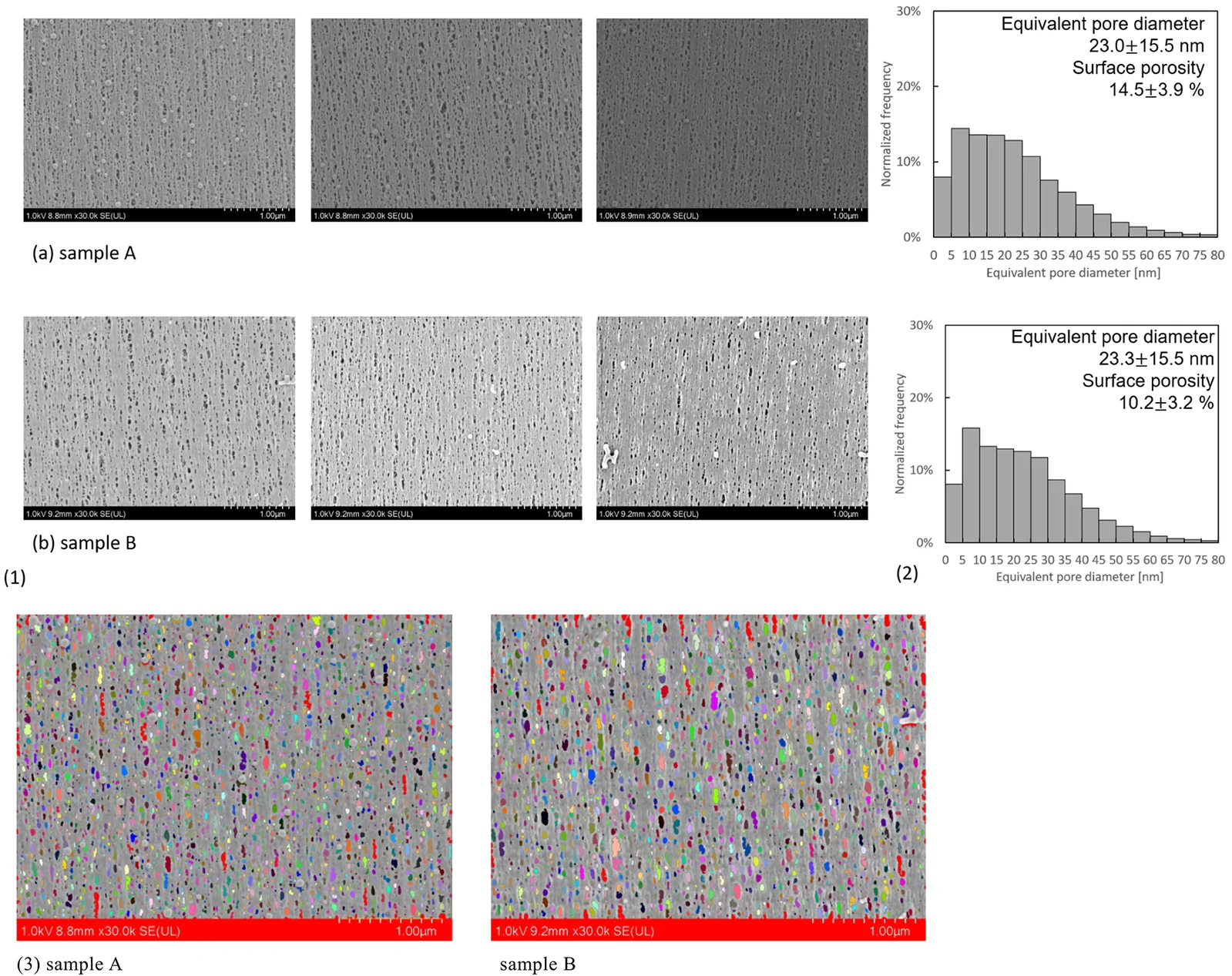
(1) FE-SEM images of the samples (A, B), inner surfaces of the capillary membrane, magnification; 30,000 (n = 3). (2) Distribution of the equivalent pore diameters of the inner lumen of the capillary membranes determined via FE-SEM. (3) Examples of an image showing the measured pore areas of samples A and B, as determined by digital image analysis

FE-SEM images of the inner surfaces of samples A and B, and the distributions of equivalent pore diameters calculated from these micrographs are shown in Fig. 5. While the pore morphology in both sample A and sample B was predominantly circular, slightly elongated pores were observed in sample B. Sample A exhibited a more porous structure, and distinct variations were noted in the morphology and frequency of granular or bean-shaped structures, which are presumed to be deposits made from PVP.

The mean equivalent pore diameters were nearly identical, with values of 23.0 ± 15.5 nm for sample A and 23.3 ± 15.5 nm for sample B. However, the surface porosity of sample A (14.5 ± 3.9%) was larger than that of sample B (10.2 ± 3.2%). These quantitative data are highly consistent with the visual observations from the FE-SEM micrographs. No statistically significant differences were found in either the equivalent pore diameter or the surface porosity.

For sample B, images of significantly higher detail than those conventionally presented for the inner membrane surface were successfully captured. Along with the findings discussed in Sect. “Correlation between the real sieving coefficient and equivalent pore diameter, significance and future challenges”, these results serve as robust data for evaluating the correlation between the membrane structure and its transport performance.

The actual processed images used for analysis are added to Fig. 5(3). The total number of pores identified across three observation fields was 3028 ± 585 for sample A and 2090 ± 455 for sample B. Under the same pore identification methodology, the higher pore count in sample A suggests that its surface porosity is greater than that of sample B; however, the resulting impact on the ${SC}_{obs}$ is expected to be minimal.

### Correlation between the real sieving coefficient and equivalent pore diameter, significance and future challenges

**Fig. 6:**
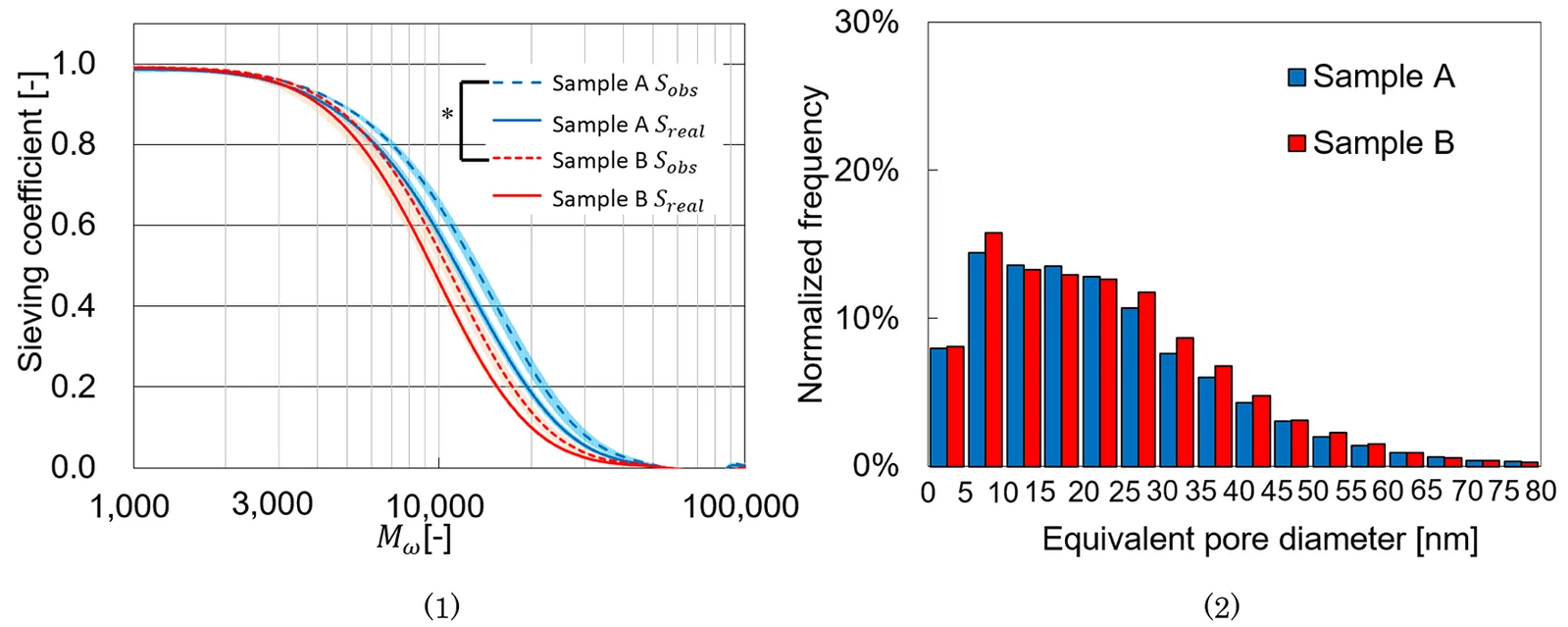
Comparison of (1) molecular weight – real and observed sieving coefficients at 60 mL/min/m^2^ and (2) equivalent pore diameter distributions for samples A and B. *Two-way analysis of variance (two-way ANOVA, *p* < 0.05)

Figure 6(1) illustrates a comparison between the measured ${SC}_{obs}$ curves and the ${SC}_{real}$ curves calculated from ${SC}_{obs}$, while Fig. 6(2) shows the comparison of their equivalent pore diameter distributions. Figure 6(2) summarizes the equivalent pore diameter distribution data presented in Fig. 5. Therefore, it does not involve any additional data processing, mathematical fitting, or transformation steps.

The sieving coefficient curves represent the mean values (n = 3), with the standard deviations indicated by the shaded regions. A statistically significant difference was observed between the ${SC}_{real}$ curves of the two samples (*p* = 0.03). In the ${SC}_{real}$ curves, sample B exhibited a slightly sharper profile for molecular weights above 4000. In this regard, the equivalent pore diameter distribution for sample B was found to be marginally sharper within the 0–30 nm range. These findings demonstrate that a precise correlation between the ${SC}_{real}$ curve and the equivalent pore diameter distribution, successfully capturing the subtle sieving coefficient and structural differences between samples A and B.

However, the Stokes diameter of albumin (MW: 66.5 kDa) is approximately 7 nm and the ${SC}_{obs}$ (or ${SC}_{real}$) for the albumin-equivalent molecular weight in Fig. 6(1) is nearly zero, the fact that pores over 10 nm account for approximately 80% in Fig. 6(2) suggests that the equivalent pore diameter derived from image analysis may be overestimated. Since Figs. 5 and 6(2) reflect data obtained from the unused membrane surfaces, caution must be exercised when evaluating the quantitative and time-dependent change’s correlation between Fig. 6(1) and (2). It would be highly valuable to investigate the effective cross-sectional pores that directly contribute to permeation, as well as the membrane surface conditions following fouling or pore blocking [14] by the dextran solution. The conformation and morphology of dextran in solution must be carefully considered.

According to the previous study [14] focusing on hemoconcentration membranes, the morphology of the inner membrane surfaces differed before and after perfusion with bovine blood. After use, the pores were almost completely fouled or blocked, resulting in an albumin SC of approximately 0.02—0.01. However, the hydraulic permeability (i.e., the sieving coefficient of water molecules) showed almost no reduction. This suggests that for smaller solutes, sub-visual pores not identifiable in FE-SEM images of the inner surface may significantly contribute to solute transport. While there are certainly challenges ahead, the first step is to examine the pore structure of the unused membrane.

Although the utility of performance evaluation using the dextran aqueous solution–GPC method has been demonstrated in the literature [6], this study expanded the molecular weight distribution of the dextran solution to a wider range. Highly precise SC curves from a small number of hollow fibers were obtained successfully by fabricating an original lab-scale hollow fiber membrane module, enabling a meaningful validation of the correlation between the SC curve and the membrane pore structure. While this study investigated commercial hemodiafiltration membranes with similar structures and performance characteristics, future work will extend this evaluation to samples with distinct transport properties and prototypes currently under development.

Solute transport through hemodiafiltration membranes is fundamentally governed by the complex three-dimensional (3D) pore architecture rather than by surface openings alone. Structural parameters essential to this transport—such as pore connectivity, tortuosity—cannot be fully characterized or quantified through two-dimensional surface FE-SEM imagery. The significant discrepancy observed between the measured equivalent pore diameters and the actual albumin rejection behavior further underscores this methodological limitation.

While obtaining a comprehensive output that encompasses all these interconnected structural factors would be ideal, it was beyond the scope of the present investigation. However, since the SC is primarily dependent on the inlet pore morphology at the membrane surface, verifying the structure of the inner membrane surface and its immediate vicinity using advanced cross-sectional techniques, such as focused ion beam scanning electron microscopy (FIB-SEM) [15] or cross-sectional ion-milling (CSIM) [13], would be highly significant for future validation. Such approaches will allow for a more rigorous understanding of the relationship between 3D membrane morphology and selective transport performance.

Meanwhile, care must be taken regarding the possibility that the dextran ${SC}_{obs}$ may be underestimated, as dextran is a linear polymer with a substantially elongated, thread-like conformation [6].

## Conclusion

Highly precise ${SC}_{obs}$ curves are successfully determined from a small number of hollow fiber membranes by utilizing the original lab-scale module and expanding the molecular weight range of the dextran aqueous solution. Reflecting the subtle structural differences between two types of commercial hemodiafiltration membranes with similar performance, a clear correlation is suggested between the ${SC}_{real}$ curves—derived from the ${SC}_{obs}$–${Q}_{F}$ relationship—and the equivalent pore diameter distribution.

Since the apparent sieving coefficient is strongly influenced by the fluid dynamics and flow path geometry within the module, evaluating the ${SC}_{real}$ is essential for directly correlating the membrane pore structure with its transport performance. Lab-scale modules are prepared relatively easily by hand, the capability to conduct rigorous and fundamental performance evaluations using this methodology is highly significant for the precise design and evaluation of advanced membranes.

## Data Availability

The data that support the findings of this study are available.

## List of symbols

*A*: Inner surface area (m^2^)
*C*: Concentration (kg/m^3^)
*D*: Inner diameter of a hollow fiber membrane (m)
f_d_: Fiber density (%)
*D*: Diffusion coefficient in water (cm^2^/s)
*D*_*h*_: Inner diameter of a housing (m)
*J*_*V*_: Filtration flux ($$\mathrm{c}\mathrm{m}/\mathrm{s})$$
*r*: Inner radius of the hollow fiber (m)
*k*: Mass transfer coefficient ($\mathrm{c}\mathrm{m}/\mathrm{s})$
*L*: Effective length of a hollow fiber (m)
*L*_*p*_: Hydraulic permeability coefficient ${\mathrm{(mL}} \cdot {\mathrm{hr}}^{{ - 1}} \cdot {\mathrm{m}}^{{ - 2}} \cdot {\mathrm{mmHg}}^{{ - 1}} )$
MW: Molecular weight (–)
N: Number of hollow fiber membranes (–)
*Q*: Flow rate (mL/min)
*R*: Rejection rate (–)
T: Temperature (K)
*V*_*F*_: Filtrated volume (mL)
${\gamma}_{w}$: Wall shear rate (${\mathrm{s}}^{-1})$
*μ *: Viscosity of water ($\mathrm{P}\mathrm{a}\bullet \mathrm{s}$)
*ΔP*: Pressure drop ($\mathrm{P}\mathrm{a}$)
*Δx*: Wall thickness of a hollow fiber membrane (m)

## Subscripts/superscripts

*B*: Blood
*D*: Dialysate
in: Inlet
out: Outlet
obs: Observed
real: Real
F, f: Filtrate-side, filtrated
